# The Role of Prophylactic Octreotide Following Pancreaticoduodenectomy to Prevent Postoperative Pancreatic Fistula: A Meta-Analysis of the Randomized Controlled Trials

**DOI:** 10.1055/s-0038-1675359

**Published:** 2018-10-18

**Authors:** Pankaj Kumar Garg, Jyoti Sharma, Ashish Jakhetiya, Nilokali Chishi

**Affiliations:** 1 Department of Surgery, University College of Medical Sciences and Guru Teg Bahadur Hospital, University of Delhi, New Delhi, Delhi, India; 2 Department of Surgical Oncology, Sawai Man Singh Medical College and Hospital, Jaipur, Rajasthan, India; 3 Department of Cancer Surgery, Vardhaman Mahaveer Medical College and Safdarjung Hospital, New Delhi, Delhi, India

**Keywords:** pancreatic cancer, pancreaticoduodenectomy, pancreatic fistula, octreotide, meta-analysis

## Abstract

**Introduction** A postoperative pancreatic fistula (POPF) is a major cause of morbidity and mortality following pancreaticoduodenectomy (PD). A pharmacologic approach using perioperative octreotide, a long-acting somatostatin analog having an inhibitory action on pancreatic exocrine secretion, was proposed to reduce the incidence of the POPF. Despite contradictory results in various randomized controlled trials (RCTs), the prophylactic octreotide has been widely used in the last two decades to reduce the POPF. The present meta-analysis aims to assess the effectiveness of the prophylactic octreotide in preventing the POPF following PD.

**Methods** A literature search was performed in the PubMed for the RCTs that compared the prophylactic octreotide with the placebo following PD published prior to October 2016. Review manager (Cochrane Collaboration's software) version RevMan 5.2 was used for analysis. Those RCTs which had compared the prophylactic Octreotide with placebo to reduce the POPF following PD were considered eligible for the meta-analysis. The low quality (Jadad score of two or less) RCTs or those including mixed pancreatic resections without reporting specific pancreaticoduodenectomy outcomes were excluded. The effect size for the dichotomous and the continuous data was displayed as the odds ratio (OR) and the weighted mean difference (WMD), respectively, with their corresponding 95% confidence intervals (CI). A fixed effect or random effects model was used to pool the data according to the result of a statistical heterogeneity test. The heterogeneity between the studies was evaluated using the Cochran Q statistic and the *I* ^2^ test, with *p* < 0.05 indicating significant heterogeneity.

**Results** There were eight RCTs available for the analysis. A total of 959 patients were included in the meta-analysis–492 received the prophylactic octreotide and 467 patients received the placebo. The prophylactic octreotide was not found to significantly decrease the total number of the POPF (OR, 1.03'; 95% CI: 0.73–1.45; *p* -value 0.85) or the clinically significant POPF (OR, 0.76; 95% CI: 0.35–1.65; *p* -value 0.49) compared with the placebo. There was also no difference in the duration of hospital stay (WMD, 1.19; 95% CI:1.84–4.23; *p* -value 0.44) or the postoperative mortality (OR, 2.04; 95% CI: 0.87–4.78; *p* -value 0.10) between the two groups. The prophylactic octreotide was also not found to significantly delay the gastric emptying (OR, 0.76; 95% CI: 0.41–1.40; *p* -value 0.38).

**Conclusion** The present meta-analysis does not support the role of the prophylactic octreotide to prevent the POPF following PD.

Pancreaticoduodenectomy (PD) is the only curative option available for the treatment of pancreatic and periampullary cancers. PD has always been considered to be associated with a high perioperative morbidly and mortality. With the advancements in the perioperative critical care and the refinements in the surgical techniques, the postoperative morbidity following PD has drastically come down to less than 5% in the high volume centers. 1 Postoperative pancreatic fistula (POPF) remains a major cause of the morbidity and the mortality following PD. Because of the lack of uniformity in the definition of the POPF, there has been a wide variation in its reported incidence ranging from 2% to more than 20% (2). In 2005, an international study group on pancreatic fistula (ISGPF), an international panel of pancreatic surgeons working in well-known high-volume centers, formulated an acceptable and an objective definition of the POPF to decrease the interobserver variability. They defined the POPF as a drain output of any measurable volume of fluid on or after the postoperative day 3 with an amylase content of greater than three times the serum amylase activity. 2

How the POPF can be prevented continues to remain a challenge for the surgeons. Several surgical modifications have been proposed to reduce the risk of the POPF: (1)the type of pancreaticointestinal anastomosis (pancreaticogastrostomy versus pancreaticojejunostomy), (2) the different methods of doing anastomosis (duct to mucosa, invagination), (3) the use of stents (internal vs. external vs. no stent), and (4) the application of various topical sealants over the anastomosis. 3 A pharmacologic approach using perioperative octreotide, a long-acting somatostatin analog having an inhibitory action on the pancreatic exocrine secretions, was also proposed to reduce the incidence of the POPF. Despite contradictory results in various randomized controlled trials (RCTs), the prophylactic octreotide has been widely used in the last two decades to reduce the POPF. The present meta-analysis aims to assess the effectiveness of the prophylactic octreotide in preventing the POPF following a PD.

## Methods

A literature search was performed in the PubMed for the RCTs that compared the prophylactic octreotide with the placebo following a PD published prior to October 2016. Review manager (Cochrane Collaboration's software) version RevMan 5.2 was used for the analysis. Those RCTs, which had compared the prophylactic octreotide with placebo to reduce the POPF following PD, were considered eligible for the meta-analysis. Low quality (Jadad score of two or less) RCTs or those including mixed pancreatic resections without reporting specific PD outcomes were excluded. The effect size for dichotomous and continuous data was displayed as odds ratio (OR) and weighted mean difference (WMD), respectively, with their corresponding 95% confidence intervals (CI). A fixed effect or random effects model was used to pool the data according to the result of a statistical heterogeneity test. Heterogeneity between studies was evaluated using the Cochran Q statistic and the *I* ^2^ test, with *p* < 0.05 indicating significant heterogeneity.

## Result

**Fig. 1:**
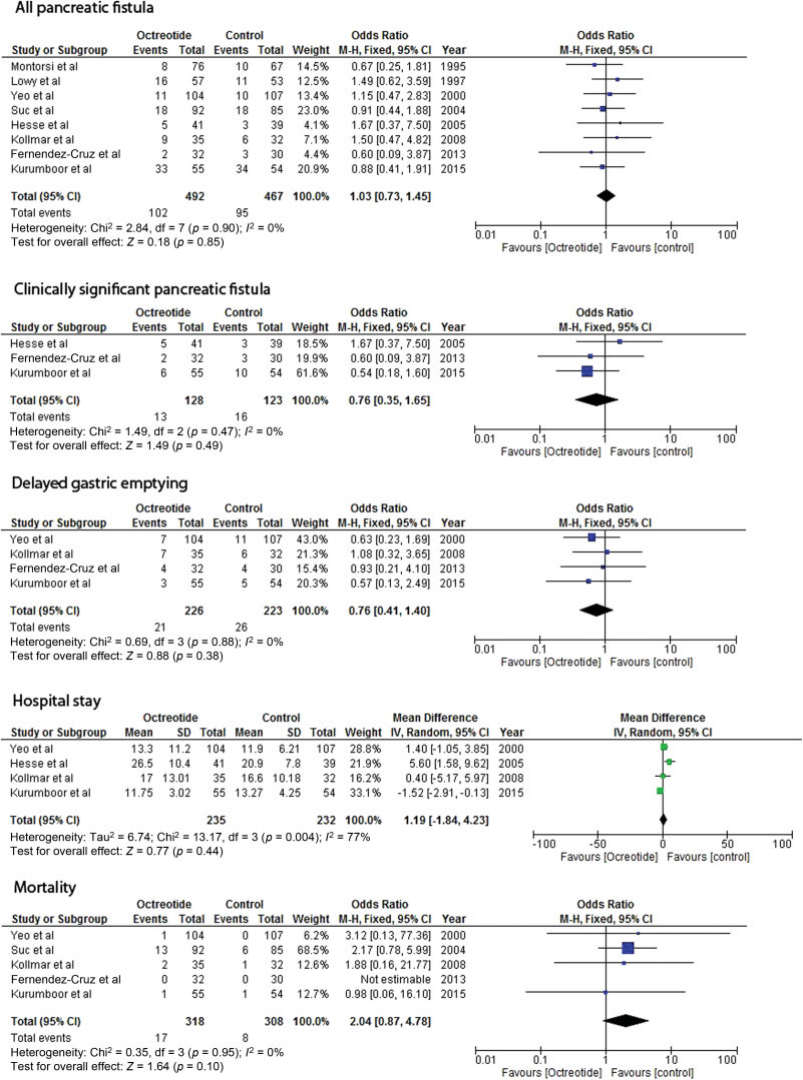
Displays the forest plots of eight RCTs comparing the prophylactic role of octreotide to prevent the POPF compared with placebo. CI, confidence interval; df, degrees of freedom; M-H, Mantel-Haenszel.

There were eight RCTs available for the analysis ( Table 1 ). 4 5 6 7 8 9 10 11 A total of 959 patients were included in the meta-analysis–492 received the prophylactic perioperative octreotide and 467 patients received the placebo. The perioperative octreotide was not found to significantly decrease the total number of the POPF (OR, 1.03; 95% CI: 0.73–1.45; *p* -value 0.85) or the clinically significant POPF (OR, 0.76; 95% CI: 0.35–1.65; *p* -value 0.49) compared with the placebo. There was also no difference in the duration of hospital stay (WMD, 1.19; 95% CI:1.84–4.23; *p* -value 0.44) or the postoperative mortality (OR, 2.04; 95% CI: 0.87–4.78; *p* -value 0.10) in two groups. The perioperative octreotide was also not found to significantly delay the gastric emptying (OR, 0.76; 95% CI: 0.41–1.40 *p* -value 0.38). Fig. 1 displays the forest plots of the eight RCTs comparing the role of the prophylactic octreotide to prevent the POPF compared with the placebo.

**Table 1:** Characteristics of the included studies

Author	Y of publication	Sample size	Jadad score	Dose and route of octreotide	Definition of the POPF	THE POPF (octreotide vs. control)
Kurumboor et al,	2015	109	3	100 microgram octreotide SC, 8 h for 5 d	As per ISGPF definition	33/55 vs. 34/54 ( *p* = 0.626)
Fernández-Cruz et al	2013	62	3	100 microgram octreotide SC, 8 h for 10 d	As per ISGPF definition	2/32 vs. 3/30, ( *p* = 0.769)
Kollmar et al	2008	65	5	100 microgram octreotide SC, 8 h for 7 d	As per ISGPF definition	9/35 vs. 6/32 ( *p* = NS)
Hesse et al	2005	80	3	100 microgram octreotide SC, 8 h for 7 d	>100 mL/day of amylase rich fluid (> 5 times upper limit of normal serum amylase after d 3, persisting after d 7 with rising temperature and preseptic condition	5/41 vs. 3/39 ( *p* = NS)
Suc et al	2004	177	4	100 microgram octreotide SC, 8 h for 10 d	Amylase rish fluid (> 4 times upper limit of normal serum amylase after d 3	18/92 vs. 18/85 ( *p* = NS)
Yeo et al	2000	211	5	250 microgram octreotide SC, 8 h for 7 d	> 50 mL/day of amylase rich fluid (> 3 times upper limit of normal serum amylase after d 10, or pancreatic anastomotic disruption demonstrated radiologically	11/104 vs. 10/107 ( *p* = NS)
Lowy et al	1997	110	3	150 microgram octreotide SC, 8 h for 5 d	Amylase rich fluid (> 2.5 times upper limit of normal serum amylase after d 3	16/57 vs. 11/53 ( *p* = 0.23)
Montorsi et al	1995	143	4	100 microgram octreotide SC, 8 h for 7 d	> 10 mL/day of amylase rich fluid (> 3 times of normal serum amylase after d 3	8/76 vs. 10/67 ( *p* = NS)

Abbreviations: ISGPF, International Study Group for Pancreatic Fistula; NS, not significant; POPF, postoperative pancreatic fistula; SC, subcutaneously.

## Discussion

The rationale for using somatostatin in the prevention of the POPF is based on their ability to decrease the output of secretions from the pancreas as a high pancreatic juice output in a soft pancreas is an important risk factor for the POPF. 12 As the half-life of the somatostatin is short at approximately 2 minutes, the synthetic analogues of the somatostatin with longer half-lives, such as octreotide, have been developed and used in pancreatic surgery in an attempt to decrease the POPF, with the hypothesis that decreased pancreatic juice secretion would allow for an improved healing of the pancreatic ductal anastomoses and consequently would decrease the leak rates. The use of the octreotide has been studied in multiple randomized prospective trials in the United States and Europe; however, the results have been contradictory.

The current meta-analysis fails to support the prophylactic role of octreotide in reducing the chances of the POPF, delayed gastric emptying (DGE), duration of hospital stay, or mortality. However, the results of this meta-analysis should be viewed in the light of potential confounding factors–a nonuniform definition of the POPF, a different dosing regimen of the octreotide employed, the surgical technique and experience of different surgeons, and other high-risk factors for the POPF. A nonuniform definition of the POPF has been a major barrier in evaluating the results of various studies addressing the issue of the POPF; however, a widespread acceptance of the ISGPF definition of the POPF has resulted in bringing some homogeneity in the studies. All three of the eight RCTs published after the year 2005 used the ISGPF definition of the POPF. Various dosing regimen is another concern while comparing the results of the prophylactic octreotide across the studies. Both the dose (100–250 micrograms) and duration (5–10 days) of the prophylactic octreotide were used by authors differently in various studies. Yeo et al 4 used a high dose of octreotide (250 micrograms three times a day) in their RCT of 211 patients. They also reported the ineffectiveness of the prophylactic octreotide following PD in reducing the POPF (9 vs. 11%), the overall complication rates (34 vs. 40%), the in-hospital death rates (0 vs. 1%), and the duration of hospital stay (9 vs. 9 days). The authors admitted that they had used a higher dose intentionally so as not be blamed for using an inadequate dose of the prophylactic octreotide had their RCT turned out to be a negative one.

Various RCTs have been criticized for not assessing the role of the prophylactic octreotide in those patients having high-risk factors for the POPF. Though several factors have been implicated in the formation of the POPF, nondilated pancreatic duct and soft pancreas are widely reported as high-risk factors for the POPF. 6 13 14 A study of 109 patients undergoing PD with soft pancreas and nondilated pancreatic duct did not show any statistically significant difference in rates of the pancreatic fistula with the use of the prophylactic octreotide (Total POPF, 60 vs. 63%). 6

Pasireotide is a newer generation long-acting somatostatin analog which is being evaluated to decrease the POPF following PD. However, the results of the prophylactic pasireotide have also been variable till now. A single-center, randomized, double-blind trial of perioperative subcutaneous pasireotide in patients undergoing PD showed a significant reduction in the rates of grade three or higher postoperative pancreatic fistula, leak, or abscess among patients who received pasireotide compared with the patients who received placebo (10 vs. 21%; relative risk, 0.49; 95% CI: 0.25–0.95). 15 However, another recently published prospective case-control study revealed no difference in the pancreatic fistula, the overall complications, the 90-day readmission or the 90-day mortality in the patients who received the pasireotide. 16 However, it would be too early to arrive to a conclusion regarding the efficacy of the prophylactic pasireotide following PD.

Interestingly, the present meta-analysis suggests a trend toward a higher mortality in the patients receiving prophylactic octreotide though it failed to attain a statistical significance (OR, 2.04; 95% CI: 0.87–4.78; *p* -value 0.10). However, this may prove to be a final nail in the coffin of use of prophylactic octreotide following PD. Several reasons may be attributed to this unexpected outcome as octreotide causes: (1) a reduction in the splanchnic blood flow; (2) suppression of the secretion of anabolic and tropic hormones such as pituitary growth hormone (GH), insulin-like growth factor 1, and epidermal growth factor; and (3) a reduction in the volume of the pancreatic juice resulting in large fluctuations in the enzyme concentration. 17

Despite many limitations, the accumulated evidence through the present meta-analysis indicates that the prophylactic octreotide has failed to show any demonstrable benefit in reducing the POPF. Moreover, elimination of the prophylactic octreotide would also be helpful as a considerable cost saving measure. The time has come to look for other newer modalities including both pharmacological and nonpharmacological to address the issue of the POPF which continues to haunt the surgeons performing PD.

## Conclusion

The present meta-analysis does not support the role of the prophylactic octreotide to prevent the POPF following PD.
